# Clinical analysis of 16 patients with *Clostridium butyricum* isolated from blood cultures: A 10-year single-center case series and review of published cases

**DOI:** 10.1016/j.nmni.2026.101821

**Published:** 2026-07-30

**Authors:** Sao Yoshinaga, Masahiro Watanabe, Hiroshi Ito, Shinji Hatakeyama, Yoshiharu Saitou, Ryuya Yamamura, Naoaki Hashimoto, Toru Nanmoku, Kiyofumi Ohkusu, Hiromichi Suzuki

**Affiliations:** a Department of Infectious Diseases, University of Tsukuba Hospital, Japan; b Clinical Laboratory Department, University of Tsukuba Hospital, Japan; c Department of Hospital Medicine, University of Tsukuba Hospital, Japan; d Department of Pharmacy, University of Tsukuba Hospital, Japan; e Department of Microbiology, Tokyo Medical University, Japan

**Keywords:** *Clostridium butyricum*, Probiotics, Blood cultures, PCR ribotyping, Case series

## Abstract

**Objectives:**

*Clostridium butyricum* is an anaerobic gram-positive bacillus widely used as a probiotic preparation in Japan. Although generally considered safe, *C. butyricum* has rarely been isolated from blood cultures, particularly in immunocompromised or medically complex patients. We aimed to describe the clinical characteristics, microbiological findings, treatment, and outcomes of patients with *C. butyricum* isolated from blood cultures at a single academic hospital and to summarize published cases.

**Methods:**

We retrospectively reviewed 16 patients with *C. butyricum* isolated from blood cultures at University of Tsukuba Hospital between 2015 and 2025. Clinical characteristics, microbiological findings, antimicrobial treatment, and outcomes were evaluated. Preserved institutional isolates were identified using mass spectrometry and polymerase chain reaction–based ribotyping. We also summarized 31 published cases descriptively.

**Results:**

Sixteen institutional cases were identified. The most common clinical characteristic was central venous catheter use (75.0%). In many cases, *C. butyricum* was isolated from a single anaerobic blood culture bottle. All preserved institutional isolates were confirmed as *C. butyricum* and showed type B ribotyping patterns; however, strain-level attribution was not possible. All patients received β-lactam antibiotics, and three patients (18.8%) died. The 31 published cases were heterogeneous in age, clinical context, and microbiological identification methods.

**Conclusions:**

*C. butyricum* is rarely isolated from blood cultures but may warrant clinical assessment in medically complex patients. A possible association with probiotic exposure requires further investigation using genomic methods.

## Highlights

• A 10-year single-center series of *C. butyricum* isolated from blood cultures is described
• Institutional isolates showed patterns similar to previously reported type B ribotyping patterns
• Most institutional cases involved medically complex or immunocompromised patients
• Single-bottle isolation may warrant clinical assessment in vulnerable hosts

## 1 Introduction

*Clostridium butyricum* is an anaerobic gram-positive bacillus that has been widely used in Japan as a probiotic preparation under trade names such as “Miya-BM.” Its efficacy in improving intestinal microbiota has been reported in conditions such as antibiotic-associated diarrhea and irritable bowel syndrome [[1], [2], [3], [4], [5]]. Adverse events associated with probiotic preparations are generally limited to mild gastrointestinal symptoms, and serious complications are rare [6]. Accordingly, *C. butyricum* has generally been regarded as a safe probiotic organism.

Microorganisms used in probiotic preparations may, in rare circumstances, be isolated from normally sterile sites, including blood cultures. Clinically meaningful bloodstream infection may occur when microorganisms translocate from colonized mucosal surfaces into the circulation, particularly in patients with impaired host defenses or disruption of mucosal barriers. Bloodstream infections associated with other probiotics, including *Lactobacillus* and *Saccharomyces* species, have been reported mainly in immunocompromised patients and those receiving intensive medical interventions [7,8]. Sporadic cases of patients with *C. butyricum* isolated from blood cultures have also been reported, although the extent to which these cases are directly related to probiotic exposure often remains uncertain.

Clinical data on patients with *C. butyricum* isolated from blood cultures remain limited, and their clinical presentation, microbiological features, treatment, and outcomes have not been well characterized. Therefore, we retrospectively analyzed patients with *C. butyricum* isolated from blood cultures at our institution and summarized published cases for descriptive context. We also evaluated the possible association with probiotic exposure while recognizing the limitations of available strain-level evidence.

## 2 Methods

This study consisted of two components: (1) a retrospective observational study conducted at a single academic hospital and (2) a descriptive summary of published cases collected for clinical context. The retrospective study included patients in whom *C*. *butyricum* was isolated from blood cultures at University of Tsukuba Hospital between 1 September 2015 and 31 August 2025. The study protocol was reviewed and approved by the Ethics Committee of University of Tsukuba Hospital (No. R07-164). The requirement for informed consent was waived by the institutional review board because of the retrospective nature of the study.

### 2.1 Clinical data collection at our hospital

We reviewed the electronic medical records of patients with *C. butyricum* isolated from blood cultures at our hospital through 31 August 2025. For this study, institutional cases were operationally defined as patients with *C. butyricum* isolated from at least one blood culture bottle. Because the clinical significance of single-bottle positivity may vary, we also collected information on the number of positive blood culture bottles, whether growth occurred in aerobic or anaerobic bottles, the presence of co-isolated organisms, and the results of follow-up blood cultures when available. These microbiological and clinical features were used to support descriptive interpretation of clinical significance, but no formal adjudication system was applied. No additional clinical exclusion criteria were applied after final species-level confirmation of *C. butyricum*.

Demographic and clinical data were collected, including age, sex, underlying conditions such as malignancy, immunocompromised status, chronic diseases, central venous catheter or peripherally inserted central catheter use, history of abdominal surgery, and other relevant clinical backgrounds. We also collected information on probiotic exposure, including the type and formulation of probiotic preparations administered, timing of administration when available, antimicrobial therapy including drug class and duration, and clinical outcomes including survival status. For patients who experienced multiple episodes of *C. butyricum* isolation from blood cultures, information from the first episode was used for the institutional case series, while subsequent episodes were noted separately.

### 2.2 Microbiological identification and characterization of *C. butyricum* isolates

Blood cultures were processed using the BacT/ALERT VIRTUO system (bioMérieux, Japan) and incubated at 35°C. Positive blood culture bottles were subcultured onto sheep blood agar and chocolate agar plates under 5% CO_2_ conditions (35°C for 20–24 h), as well as anaerobic blood agar plates (Anaero Columbia RS; 35°C for 24–48 h under anaerobic conditions). Colonies grown on anaerobic plates were further subcultured to obtain pure isolates, and anaerobic growth was confirmed by comparing growth under aerobic and anaerobic conditions.

Bacterial isolates derived from positive blood culture bottles were identified using matrix-assisted laser desorption/ionization time-of-flight mass spectrometry with the VITEK MS PRIME system (bioMérieux, France). Other bacterial species isolated from blood cultures were identified using the same system after subculture on appropriate media. During the study period, blood culture isolates initially reported as *C. butyricum*, *Clostridium* spp., or anaerobic Gram-positive bacillus were reviewed to ascertain institutional cases. Preserved candidate isolates initially reported as *Clostridium* spp. or anaerobic Gram-positive bacillus were retrieved, when available, and reevaluated for species identification. The institutional isolate ascertainment process, including initial laboratory reporting, availability of preserved isolates, reevaluation, final species-level identification, and exclusion after reevaluation, is summarized in Supplementary Fig. S1.

After species identification, preserved *C. butyricum* isolates were further characterized using polymerase chain reaction (PCR) targeting the 16S–23S rDNA intergenic spacer region, as previously described [9]. Primer sets CBISF–CBISR and B2F–CB23R were used for PCR ribotyping. Type B classification was determined based on PCR amplification using the type B–specific primer B2F, with isolates showing bands of identical size to the positive control. This PCR ribotyping approach was used only for type-level characterization and comparison with previously reported type B ribotyping patterns; it was not used as evidence of strain-level source attribution to MIYAIRI-588/CBM588. Whole-genome sequencing was not performed.

Antimicrobial susceptibility testing was performed to determine minimum inhibitory concentrations for penicillin G, ampicillin, piperacillin, piperacillin/tazobactam, ampicillin/sulbactam, amoxicillin/clavulanic acid, ceftazidime, ceftriaxone, cefepime, cefmetazole, imipenem/cilastatin, meropenem, clindamycin, minocycline, cefoperazone/sulbactam, flomoxef, moxifloxacin, and metronidazole, according to Clinical and Laboratory Standards Institute guidelines [10].

### 2.3 Literature review and case extraction

We performed a literature search using PubMed, the Cochrane Library, Web of Science, and Ichushi, a Japanese medical literature database with limited online availability, with the keywords “*Clostridium butyricum*” and “bacteremia.” The search covered reports published up to 31 August 2025 (see web-only Supplementary Table S1). The search strategy was developed with reference to a previous literature review on probiotic-associated *C. butyricum* isolation from blood cultures [11].

Two researchers (S.Y. and H.I.) independently screened titles and abstracts and reviewed the full texts of potentially eligible reports. Disagreements regarding study selection were resolved through discussion with a third researcher (H.S.). We included individual cases in which *C. butyricum* was isolated from blood cultures and sufficient case-level clinical information was available. Conference abstracts were eligible when sufficient individual case-level information was available. Reports without individual patient-level data, non-human studies, review articles without extractable original cases, and duplicate reports were excluded. Duplicate reports were assessed by comparing author names, publication year, patient demographics, underlying diseases, clinical course, and reported microbiological findings. The literature-derived cases were summarized only as descriptive context because of heterogeneity in age, clinical background, publication type, microbiological identification methods, strain evidence, and reporting completeness.

### 2.4 Statistical analysis

Clinical characteristics of institutional cases were summarized using descriptive statistics. Continuous variables are presented as means with standard deviations or medians with interquartile ranges, as appropriate, and categorical variables are presented as counts and percentages. Published cases were summarized separately and descriptively. We did not perform mortality-related statistical comparisons using a combined dataset of institutional and published cases, because the published cases did not arise from a common sampling frame and included heterogeneous case reports, small case series, conference abstracts, pediatric and adult patients, variable microbiological identification methods, and incomplete clinical information. Therefore, the literature-derived cases were not used for pooled clinical or prognostic inference. All analyses were performed using Stata version 15 (StataCorp LLC, College Station, TX, USA).

## 3 Results

During the study period, nine blood culture isolates were initially reported as *C. butyricum*. In addition, 21 isolates were initially reported as *Clostridium* spp. and 79 isolates as anaerobic Gram-positive bacillus. All 100 preserved candidate isolates in the latter two categories were available for reevaluation and underwent species-level reevaluation. Seven of these 100 isolates were finally identified as *C. butyricum*, whereas 93 were not identified as *C. butyricum* and were excluded. Thus, a total of 16 institutional patients with *C. butyricum* isolated from blood cultures were included in the case series. The institutional isolate ascertainment process is shown in Supplementary Fig. S1. All 16 preserved institutional isolates were confirmed as *C. butyricum* and were classified as type B by PCR ribotyping. The ribotyping patterns were similar to previously reported type B ribotyping patterns; however, this analysis did not provide definitive strain-level attribution (see web-only Supplementary Table S2 and Supplementary Fig. S2) [12].

### 3.1 Clinical characteristics of patients with *C. butyricum* isolated from blood cultures at our hospital

**Fig. 1:**
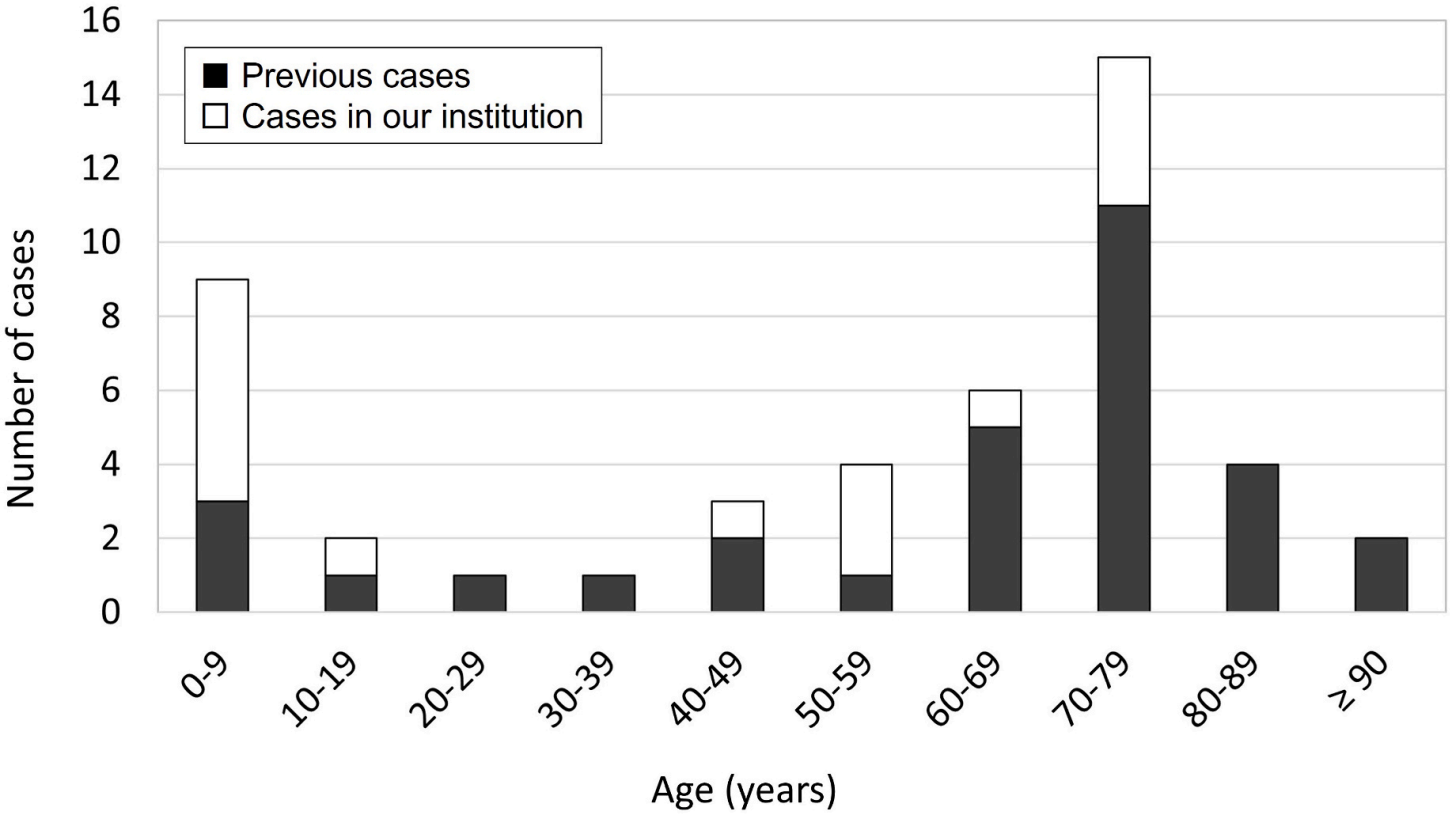
Age distribution of patients with *Clostridium butyricum* isolated from blood cultures.

Among the 16 patients identified at our hospital, the mean age was 37.9 years (standard deviation [SD], 30.2), and 11 patients (68.8%) were male (Table 1). The age distribution was bimodal (Fig. 1). The most common underlying condition was the presence of a central venous catheter (12 patients, 75.0%), followed by solid tumors (5 patients, 31.3%). Documented exposure to *C. butyricum* probiotic preparations was present in 14 patients (87.5%).

**Table 1:** Baseline characteristics of patients with *Clostridium butyricum* isolated from blood cultures at our hospital.

No	Age, years	Sex	Diagnosis on admission	Underlying diseases	Hospital day at onset	Days from probiotic initiation to onset	Probiotics form	EN	CVC/PICC
1	74	M	Perianal abscess	Ulcerative colitis (post total colectomy), lung cancer, Parkinson disease	0	16	granule	Y	N
2	48	F	Acute myeloid leukemia (relapsed after cord blood transplantation)	Intestinal emphysema, end-stage kidney disease on dialysis, schizophrenia	54	111	granule	Y	Y
3	2	F	Small for gestational age	Ileal perforation, Adhesive intestinal obstruction, short bowel syndrome	830	610	granule	Y	Y
4	3	M	Chronic subdural hematoma	Abuse, primary intracranial tumor	123	39	granule	N	Y
5	17	M	Acute subdural hematoma	Brain abscess	219	149	granule	Y	N
6	1	M	Atypical teratoid rhabdoid tumor	-	126	87	granule	Y	Y
7	78	M	Febrile neutropenia	DM, bladder cancer, encephalitis	55	47	granule	Y	Y
8	53	M	Skin and soft tissue infection	-	23	12	granule	N	Y
9	60	F	Asthma	Hypertension, DM	13	11	granule	Y	Y
10^a^	2	M	T-cell leukemia	-	136	NA	NA	Y	Y
11^a^	7	M	Pleomorphic rhabdomyosarcoma	Bulbar spinal muscular atrophy, attention-deficit/hyperactivity disorder	54	NA	NA	N	Y
12	71	M	Diffuse large B-cell lymphoma	Abdominal aortic aneurysm, duodenal stenosis, hemorrhagic gastritis	24	19	granule	N	Y
13	1	M	Acute myeloid leukemia	Neonatal jaundice	31	21	granule	Y	Y
14	58	F	Caffeine toxicity	Breast cancer	7	3	granule	Y	N
15	74	F	Hypercalcemia, Hypokalemia	Atypical lung carcinoid, DM, DL	1	365	granule	N	Y
16	57	M	Dural arteriovenous fistula, pneumonia	Hypertension, DM, DL, depression	31	6	granule	Y	N

NA indicates information not available in the retrospective medical records unless otherwise specified.

CVC, central venous catheter; DM, diabetes mellitus; DL, dyslipidemia; EN, enteral nutrition; NA, not available; N, no; PICC, peripherally inserted central catheter; Y, yes.

a Probiotic exposure and formulation could not be ascertained in Cases 10 and 11.

*C. butyricum* was isolated from a single blood culture bottle in 12 patients (75.0%), all of which involved anaerobic bottles (Table 2). Other bacterial species were co-isolated from blood cultures in four patients. Follow-up blood cultures were obtained in 13 patients, and persistent isolation of *C. butyricum* was observed in one of these patients (1/13, 7.7%). One patient experienced a second episode of *C. butyricum* isolation from blood cultures 1 year after the first episode. All patients received β-lactam antibiotics. No distant infectious foci clearly attributable to *C. butyricum* infection were identified. Three patients (18.8%) died.

**Table 2:** Clinical features, microbiological findings, and treatment of patients with *Clostridium butyricum* isolated from blood cultures at our hospital.

No	Symptoms at blood culture	No. of BC bottles positive for *C. butyricum*/total bottles^a^	Co-isolated organisms in blood cultures	Antimicrobial agents administered during the clinical course	Antimicrobial duration, days	Follow-up BC positive for *C. butyricum*	Survival at discharge
1	Fever	2/4	None	MEPM, ABPC/SBT, AMPC/CVA^b^	20	NA^d^	Y
2	Fever	1/4	*Staphylococcus warnei*	MEPM, VCM, FLCZ	NA	NA^d^	N
3	Fever, vomiting, diarrhea	2/6	None	CMZ	20	N	Y
4	Fever	1/4	None	CZOP, TAZ/PIPC, MEPM	NA	N	Y
5	Vomiting	1/4	None	MEPM	5	N	Y
6	Fever	1/6	None	CFPM	NA	N	Y
7	Fever	1/4	None	MEPM, VCM, VLCZ, GCV	NA	N	N
8	Stomachache	2/4	None	LVFX, TAZ/PIPC	NA	N	Y
9	Fever	1/4	None	ABPC/SBT	14	N	Y
10	Fever	1/2	None	CZOP, MEPM	NA	N	Y
11	Fever	1/8	*Bacillus* spp.	ABPC/SBT, MEPM, VCM	10	N	Y
12	Fever	1/4	None	CFPM, MCFG	NA	Y	N
13	Fever	1/4	None	CFPM, TAZ/PIPC	14	N	Y
14	Fever	2/4	*Staphylococcus aureus*^c^	TAZ/PIPC, CEZ	NA	N	Y
15	Impaired mobility	1/4	Unidentified Gram-positive rods	SBT/CPZ	22	N	Y
16	Fever, diarrhea	1/4	None	MEPM, ABPC/SBT	10	NA****	Y

NA indicates information not available in the retrospective medical records unless otherwise specified.

ABPC, ampicillin; AMPC/CVA, amoxicillin/clavulanic acid; BC, blood culture; CEZ, cefazolin; CFPM, cefepime; CMZ, cefmetazole; CPZ, cefoperazone; CZOP, cefozopran; GCV, ganciclovir; FLCZ, fluconazole; LVFX, levofloxacin; MCFG, micafungin; MEPM, meropenem; N, no; NA, not available; PIPC/TAZ, piperacillin/tazobactam; SBT, sulbactam; VCM, vancomycin; VLCZ, voriconazole; Y, yes.

a In cases in which only one blood culture bottle was positive for *C. butyricum*, growth was observed in an anaerobic bottle.

b All antimicrobial agents were administered intravenously, except for AMPC/CVA, which was administered orally.

c In Case 14, methicillin-susceptible *S*. *aureus* was co-isolated from all four blood culture bottles; therefore, the clinical contribution of *C. butyricum* was uncertain.

d NA for follow-up blood culture indicates that no repeat blood cultures were documented in the available medical records.

### 3.2 Descriptive summary of published cases

The literature search identified six reports from PubMed, six from Web of Science, none from the Cochrane Library, and 17 from Ichushi that described patients with *C. butyricum* isolated from blood cultures (see web-only Supplementary Table S3 and Supplementary Fig. S3). After excluding duplicate reports and cases not involving *C. butyricum* isolation from blood cultures, 31 published cases were included in the literature review. These published cases were summarized only as descriptive context and were not used for pooled clinical or prognostic inference.

The published cases were heterogeneous in age, clinical background, publication type, microbiological identification methods, strain evidence, treatment information, and outcome reporting (Fig. 1, Supplementary Table S3). Because of this heterogeneity, mortality-related statistical comparisons combining institutional and published cases were not performed.

## 4 Discussion

We identified 16 patients with *C. butyricum* isolated from blood cultures over a 10-year period at our institution, representing a large single-center series of this rare microbiological finding. The affected patients were frequently medically complex, with backgrounds such as central venous catheter use, immunocompromised status, or underlying malignancies. Published cases were summarized separately as descriptive context because of heterogeneity in age, clinical background, microbiological identification methods, strain evidence, and reporting completeness. Therefore, we did not use the literature-derived cases for pooled clinical or prognostic inference.

In Japan, *C. butyricum* MIYAIRI-588 (commercially marketed as “Miya-BM”) has been widely prescribed for decades as an approved probiotic medication to improve digestive symptoms and prevent antibiotic-associated diarrhea [13], and its role in modulating the gut microbiota has been documented [5]. In our institutional cohort, most, but not all, patients had documented exposure to *C. butyricum* probiotic preparations. Preserved institutional isolates showed PCR ribotyping patterns similar to previously reported type B ribotyping patterns. However, because PCR ribotyping provides only type-level characterization, these findings do not establish that the isolates originated from commercial probiotic preparations, including CBM588/MIYAIRI-588. Therefore, any relationship between probiotic exposure and blood culture isolates remains speculative and requires confirmation using more discriminatory genomic methods. The rarity of reported *C. butyricum* isolation from blood cultures despite widespread probiotic use suggests that exposure alone is unlikely to be sufficient for bloodstream invasion. Additional host or clinical factors, such as mucosal barrier disruption, severe underlying disease, antimicrobial pressure, impaired host defenses, or intravascular devices, may be required. Previously published cases also often lacked detailed information on probiotic exposure and microbiological typing, limiting classification as probiotic-associated infections.

Although *C. butyricum* is generally regarded as having low pathogenic potential, its biological characteristics may help explain occasional bloodstream isolation in vulnerable hosts. Spore formation enables survival in harsh gastrointestinal environments and may allow prolonged intestinal persistence [14]. In patients with mucosal barrier disruption, such as after surgery, chemotherapy, or critical illness, intestinal organisms may translocate into the bloodstream. Impaired host defenses may then reduce microbial clearance and contribute to clinically significant infection in selected patients [15]. These mechanisms are biologically plausible but were not directly demonstrated in the present study.

In our institutional cohort, *C. butyricum* was isolated from a single anaerobic blood culture bottle in most patients, highlighting the difficulty of distinguishing clinically significant bacteremia from contamination or transient bloodstream invasion. The clinical significance of anaerobic organisms isolated from a single blood culture bottle can be difficult to interpret [16], and similar uncertainty has been described for other *Clostridium* species, including *C. cadaveris* and *C. innocuum*, particularly in immunocompromised or postoperative patients [17,18]. These observations suggest that single-bottle isolation may warrant clinical assessment rather than automatic dismissal as contamination in vulnerable hosts. However, because several patients had other bacterial species isolated from blood cultures, the contribution of *C. butyricum* to the clinical course could not be determined with certainty in all cases. Accordingly, these findings should not be automatically interpreted as probiotic-induced sepsis; rather, in some patients, they may have represented transient mucosal translocation, clinically uncertain bloodstream invasion, or blood culture positivity of unclear significance in highly vulnerable, multimorbid hosts.

All patients in our cohort received β-lactam antibiotics during the clinical course. This treatment pattern was consistent with previous reports, in which β-lactam agents were commonly used. Available susceptibility data suggest that several β-lactams may have activity against *C. butyricum*, although resistance to penicillin G, amoxicillin, and piperacillin has been reported in some strains [19]. A β-lactamase–producing strain has also been described, although several β-lactams retained activity [20]. Overall, β-lactams may be reasonable treatment options when antimicrobial therapy is clinically indicated, but optimal treatment has not been established.

Bloodstream infections associated with other probiotic organisms, such as *Lactobacillus* and *Saccharomyces* species, have also been reported mainly in patients with immunocompromised status, critical illness, broad-spectrum antimicrobial exposure, or indwelling catheters [[21], [22], [23], [24]]. These host factors overlap with those observed in our institutional series. However, the microbiological characteristics, routes of bloodstream invasion, and certainty of probiotic attribution differ among organisms and reports. Therefore, our findings should be interpreted as consistent with the broader literature on probiotic-associated invasive infections, rather than as direct evidence that *C. butyricum* isolation from blood cultures follows the same pathogenesis or prognosis.

This study has several limitations. First, although institutional isolates were identified as *C. butyricum* using mass spectrometry and further characterized by PCR ribotyping, whole-genome sequencing was not performed. Therefore, definitive strain-level attribution to MIYAIRI-588/CBM588 could not be established. Second, documented probiotic exposure was not confirmed in all institutional cases, and information on probiotic exposure was often incomplete in previously published cases. Thus, the relationship between probiotic preparations and *C. butyricum* isolation from blood cultures should be regarded as a speculative hypothesis rather than evidence of causality or strain-level attribution. Third, *C. butyricum* was isolated from a single anaerobic blood culture bottle in many institutional cases, and some patients had other bacterial species isolated from blood cultures. Therefore, contamination, transient bacteremia, or polymicrobial bacteremia could not be excluded in all cases, and the clinical contribution of *C. butyricum* could not always be determined with certainty. Inflammatory markers and sepsis severity scores were not uniformly available and therefore could not be systematically assessed. Fourth, previously published cases were derived from case reports and small case series, which were subject to incomplete clinical documentation, variable microbiological identification methods, and reporting bias. In addition, the combined dataset included both pediatric and adult patients, whose underlying conditions and clinical contexts may differ substantially. For these reasons, mortality-related statistical comparisons combining institutional and published cases were not performed. Finally, because of the limited sample size and heterogeneity of included cases, multivariable analysis could not be performed.

In conclusion, *C. butyricum* isolation from blood cultures is rare but may be clinically relevant in medically complex or immunocompromised patients. Single-bottle positivity and co-isolated organisms may complicate interpretation, and the clinical contribution of *C. butyricum* cannot always be determined with certainty. PCR ribotyping provided type-level characterization but did not allow strain-level attribution to probiotic strains. Published cases should be interpreted as descriptive context because of substantial clinical and microbiological heterogeneity. Further genomic studies are needed to clarify the relationship between probiotic exposure and bloodstream isolates.

## Data availability statement

The data supporting the findings of this study are available from the corresponding author upon reasonable request, subject to institutional and ethical restrictions.

## Generative AI and AI-assisted technologies statement

During the preparation of this work, the authors used ChatGPT 5.5 (OpenAI) to assist in preparing an English draft and refining wording before professional English language editing by *Editage* (www.editage.com). The authors did not use this tool to generate scientific content, analyze data, or draw conclusions. After using this tool, the authors reviewed and edited the content as needed and take full responsibility for the content of the published article.

## Funding

None to declare.

## CRediT authorship contribution statement

**Sao Yoshinaga:** Data curation, Formal analysis, Visualization, Writing – original draft. **Masahiro Watanabe:** Data curation, Investigation, Visualization. **Hiroshi Ito:** Project administration, Visualization, Writing – original draft. **Shinji Hatakeyama:** Investigation, Writing – review & editing. **Yoshiharu Saitou:** Investigation, Writing – review & editing. **Ryuya Yamamura:** Investigation, Writing – review & editing. **Naoaki Hashimoto:** Data curation, Writing – review & editing. **Toru Nanmoku:** Investigation, Writing – review & editing. **Kiyofumi Ohkusu:** Investigation, Supervision, Writing – review & editing. **Hiromichi Suzuki:** Project administration, Writing – review & editing.

## Declaration of competing interest

The authors declare that they have no known competing financial interests or personal relationships that could have appeared to influence the work reported in this paper.
