# Supplementary material for: Clinical analysis of 16 patients with Clostridium butyricum isolated from blood cultures: A 10-year single-center case series and review of published cases
Source: New Microbes New Infect. 2026 Jul 30;73:101821. doi: 10.1016/j.nmni.2026.101821 (PMC13453462; doi:10.1016/j.nmni.2026.101821)
Supplement: Supplementary file 1 [file mmc1.docx]

**Table S1. Search strategies for reports of *Clostridium butyricum* isolated from blood cultures**

PubMed

(((("Clostridium butyricum"[Text Word] OR "c.butyricum"[Title/Abstract]) AND ("Infections"[MeSH Terms] OR "Bacterial Infections"[MeSH Terms] OR "Communicable Diseases"[MeSH Terms] OR ("Bacteremia"[MeSH Terms] OR ("bacteremia*"[Title/Abstract] OR "bacteraemia*"[Title/Abstract]) OR "septicemia*"[Text Word]))) OR (("Clostridium butyricum"[Text Word] OR "c.butyricum"[Title/Abstract]) AND "review"[Text Word])) NOT ("animals"[MeSH Terms] NOT "humans"[MeSH Terms])) AND ("English"[Language] OR "Japanese"[Language])

Web of Science

#1 TS = ("Clostridium butyricum" OR "c.butyricum")

#2 TS = ("Infections" OR "Bacterial Infections" OR "Communicable Diseases" OR "Bacteremia" OR bacteremia* OR bacteraemia* OR septicemia*)

#3 TS = (animal* OR mice OR mouse OR rat* OR murine OR rabbit* OR canine* OR feline* OR swine* OR porcine* OR primate* OR zebrafish OR drosophila)

#4 TS = (human* OR patient* OR adult* OR child* OR infant* OR neonat*)

#1 AND #2 NOT (#3 NOT #4) AND LA = (English OR Japanese)

Cochrane Library

#1 "Clostridium butyricum":ti,ab,kw OR "c.butyricum":ti,ab,kw

#2 MeSH descriptor: [Infections] explode all trees

#3 MeSH descriptor: [Bacterial Infections] explode all trees

#4 MeSH descriptor: [Communicable Diseases] explode all trees

#5 MeSH descriptor: [Bacteremia] explode all trees

#6 bacteremia*:ti,ab,kw OR bacteraemia*:ti,ab,kw OR septicemia*:ti,ab,kw

#7 review:ti,ab,kw

#8 MeSH descriptor: [Animals] explode all trees

#9 MeSH descriptor: [Humans] explode all trees

#1 AND (#2 OR #3 OR #4 OR #5 OR #6) OR (#1 AND #7) NOT (#8 NOT #9) AND english:la

Ichushi

#1 "Clostridium butyricum"/AL OR "C. butyricum"/AL OR Amylobacter/TA

#2 Miyairi/TA OR Miyairi-kin/TA OR Miyarisan/TA OR butyric acid producing bacillus/TA

#3 probiotics/TH AND SH=toxicity OR SH=”adverse events”

#4 infection/TH OR bacterial infections/TH OR bacteremia/TA OR septicemia/TA

(#1 OR #2 OR #3) AND #4 NOT (CK = animals NOT CK = humans)

**Table S2. Minimum inhibitory concentrations of *Clostridium butyricum* isolates from blood cultures at our hospital**

| No | PCG | ABPC | PIPC | PIPC/TAZ | ABPC/SBT | AMPC/CVA | CAZ | CTRX | CFPM | CMZ | IPM/CS | MEPM | CLDM | MINO | CPZ/SBT | FMOX | MFLX | MNZ |
| --- | --- | --- | --- | --- | --- | --- | --- | --- | --- | --- | --- | --- | --- | --- | --- | --- | --- | --- |
| 1 | 0.25 | ≤0.12 | ≤2 | ≤2 | ≤2 | ≤1 | >32 | 8 | >32 | ≤1 | 0.5 | ≤0.25 | ≤0.12 | ≤0.25 | ≤4 | ≤1 | 0.5 | ≤2 |
| 2 | 0.25 | 0.25 | ≤2 | ≤2 | ≤2 | ≤1 | >32 | 8 | >32 | 2 | ≤0.25 | ≤0.25 | ≤0.12 | ≤0.25 | ≤4 | ≤1 | 0.5 | ≤2 |
| 3 | 0.25 | ≤0.12 | ≤2 | ≤2 | ≤2 | ≤1 | >32 | 8 | 32 | ≤1 | ≤0.25 | ≤0.25 | ≤0.12 | ≤0.25 | ≤4 | ≤1 | 0.5 | ≤2 |
| 4 | 0.25 | ≤0.12 | ≤2 | ≤2 | ≤2 | ≤1 | >32 | 8 | >32 | ≤1 | ≤0.25 | ≤0.25 | ≤0.12 | ≤0.25 | ≤4 | ≤1 | 0.5 | ≤2 |
| 5 | 0.25 | ≤0.12 | ≤2 | ≤2 | ≤2 | 2 | >32 | 8 | 32 | ≤1 | 0.5 | ≤0.25 | ≤0.12 | ≤0.25 | ≤4 | ≤1 | 0.5 | ≤2 |
| 6 | 0.25 | 0.25 | ≤2 | ≤2 | ≤2 | ≤1 | >32 | 8 | >32 | 2 | 0.5 | ≤0.25 | ≤0.12 | ≤0.25 | ≤4 | ≤1 | ≤0.25 | ≤2 |
| 7 | 0.5 | 0.25 | ≤2 | ≤2 | ≤2 | ≤1 | >32 | 8 | >32 | 2 | 0.5 | ≤0.25 | 0.5 | ≤0.25 | ≤4 | ≤1 | 0.5 | ≤2 |
| 8 | 0.25 | 0.25 | ≤2 | ≤2 | ≤2 | ≤1 | >32 | 8 | >32 | 2 | 1 | ≤0.25 | 0.25 | ≤0.25 | ≤4 | ≤1 | 0.5 | ≤2 |
| 9 | 0.25 | 0.25 | ≤2 | ≤2 | ≤2 | ≤1 | >32 | 4 | >32 | 2 | 0.5 | ≤0.25 | 0.25 | ≤0.25 | ≤4 | ≤1 | ≤0.25 | ≤2 |
| 10 | ≤0.12 | ≤0.12 | ≤2 | ≤2 | ≤2 | ≤1 | >32 | 8 | 32 | 2 | 0.5 | ≤0.25 | ≤0.12 | ≤0.25 | ≤4 | ≤1 | ≤0.25 | ≤2 |
| 11 | 0.25 | ≤0.12 | ≤2 | ≤2 | ≤2 | ≤1 | >32 | 4 | >32 | ≤1 | ≤0.25 | ≤0.25 | ≤0.12 | ≤0.25 | ≤4 | ≤1 | 0.5 | ≤2 |
| 12 | 0.25 | ≤0.12 | ≤2 | ≤2 | ≤2 | ≤1 | >32 | 4 | 32 | 2 | 0.5 | ≤0.25 | ≤0.12 | ≤0.25 | ≤4 | ≤1 | 0.5 | ≤2 |
| 13 | 0.25 | 0.25 | ≤2 | ≤2 | ≤2 | ≤1 | >32 | 4 | >32 | 2 | ≤0.25 | ≤0.25 | ≤0.12 | ≤0.25 | ≤4 | ≤1 | 0.5 | ≤2 |
| 14 | 0.25 | 0.25 | ≤2 | ≤2 | ≤2 | ≤1 | >32 | 8 | >32 | ≤1 | 0.5 | ≤0.25 | ≤0.12 | ≤0.25 | ≤4 | ≤1 | 0.5 | ≤2 |
| 15 | 0.25 | ≤0.12 | ≤2 | ≤2 | ≤2 | ≤1 | >32 | 4 | >32 | 2 | 0.5 | ≤0.25 | ≤0.12 | ≤0.25 | ≤4 | ≤1 | 0.5 | ≤2 |
| 16 | 0.5 | 0.25 | ≤2 | ≤2 | ≤2 | ≤1 | >32 | 4 | >32 | 2 | 1 | ≤0.25 | 0.25 | ≤0.25 | ≤4 | ≤1 | 0.5 | ≤2 |

The minimum inhibitory concentrations are presented in μg/mL.

ABPC, ampicillin; ABPC/SBT, ampicillin/sulbactam; AMPC/CVA, amoxicillin/clavulanic acid; CAZ, ceftazidime; CFPM, cefepime; CLDM, clindamycin; CMZ, cefmetazole; CPZ/SBT, cefoperazone/sulbactam; CTRX, ceftriaxone; FMOX, flomoxef; IPM/CS, imipenem/cilastatin; MEPM, meropenem; MFLX, moxifloxacin; MINO, minocycline; MNZ, metronidazole; PCG, penicillin G; PIPC, piperacillin; PIPC/TAZ, piperacillin/tazobactam.

**Table S3. Clinical and microbiological characteristics of previously published cases of *Clostridium butyricum* isolated from blood cultures**

| Case  No. | Author, year | Source/publication type | Age/sex | Major clinical background | Probiotic exposure | Identification/strain evidence | Treatment | Outcome |
| --- | --- | --- | --- | --- | --- | --- | --- | --- |
| 1 | Gardner 2008 | PubMed/WoS; full article | 47 / M | rheumatoid arthritis, intravenous drug user, *Staphylococcus aureus* bacteremia, CVC | No | NR | CFPM, VCM, LVFX | Survived |
| 2 | Komori 2014 | Ichushi; conference abstract | 62 / M | cirrhosis, gastric ulcer, gastric cancer, DM | Yes | NR | NR | Died |
| 3 | Kondo 2017 | Ichushi; conference abstract | 83 / F | acute cholangitis | Yes | NR | NR | Survived |
| 4 | Sato 2018 | PubMed/WoS; full article | 84 / M | atrial fibrillation, stroke, abdominal penetrating injury (suicide attempt) | NR | NR | MEPM, CFPM, MNZ, CLDM | Survived |
| 5 | Hiraiwa 2020 | Ichushi; conference abstract | 72 / M | NR | Yes | NR | NR | Survived |
| 6 | Miyake 2020 | Ichushi; conference abstract | 78 / M | COPD | Yes | NR | PIPC/TAZ, ABPC/SBT | Survived |
| 7 | Hori 2020 | Ichushi; conference abstract | 98 / F | HF, stroke, pneumonia, superior mesenteric artery occlusion | Yes | NR | NR | Died |
| 8 | Hori 2020 | Ichushi; conference abstract | 72 / M | pneumococcal pneumonia | Yes | NR | ABPC/SBT | Survived |
| 9 | Shimura 2021 | PubMed/WoS; full article | 74 / M | gallbladder cancer | Yes | NR | PIPC/TAZ, VCM, DAP | Died |
| 10 | Shimura 2021 | PubMed/WoS; full article | 63 / M | cholangiocarcinoma, DM | Yes | NR | MEPM, ABPC/SBT, LVFX, VCM, MNZ | Survived |
| 11 | Ogawa 2021 | Ichushi; Japanese case report | 76 / F | mitral valve regurgitation (mitral valve replacement surgery), NOMI | Yes | NR | MEPM | Died |
| 12* | Oyabu 2021 | Ichushi; conference abstract | 43 / M | liver transplantation for Budd-Chiari syndrome | Yes | NR | MEPM, TEIC, MCFG | Survived |
| 13 | Ito 2022 | Ichushi; Japanese case report | 80s / M | hypothermia | Yes | RAPD | PIPC/TAZ | Survived |
| 14 | Watanabe 2022 | Ichushi; conference abstract | 2 / M | leukemia, febrile neutropenia | Yes | NR | NR | Survived |
| 15 | Sanemoto 2022 | Ichushi; conference abstract | 0 / F | myotonic dystrophy, necrotizing enterocolitis | Yes | Tm mapping | MEPM, VCM, ABPC/SBT | Survived |
| 16 | Ishikawa 2023 | PubMed/WoS; full article | 62 / F | eating disorder, chronic kidney disease | Yes | MALDI-TOF MS | CMZ, CFPM, CLDM, ABPC/SBT, LVFX | Survived |
| 17 | Ishikawa 2023 | PubMed/WoS; full article | 72 / F | DM, intracranial hemorrhage, hyperosmolar hyperglycemic state | Yes | MALDI-TOF MS | MEPM, CFPM, MNZ | Survived |
| 18 | Ishikawa 2023 | PubMed/WoS; full article | 76 / M | *Clostridioides difficile* infection, HF, stroke, hemodialysis, coronary artery disease | Yes | MALDI-TOF MS | PIPC/TAZ, CLDM, CFPM, MNZ, VCM | Died |
| 19 | Hayakawa 2023 | Ichushi; conference abstract | 0 / NR | trisomy 21, liver dysfunction | Yes | NR | Not reported | Survived |
| 20 | Kusudo 2023 | Ichushi; Japanese case report | 76 / F | upper gastrointestinal perforation | Yes | NR | NR | Survived |
| 21 | Imado 2023 | Ichushi; conference abstract | 60s / M | hepatocellular carcinoma, nonalcoholic steatohepatitis, DM, hypertension | Yes | NR | NR | Survived |
| 22 | Kojima 2023 | Ichushi; conference abstract | 31 / M | NR | Yes | WGS | NR | Died |
| 23 | Sanada 2023 | Ichushi; conference abstract | 79 / F | NR | No | NR | NR | Died |
| 24 | Toyama 2023 | Ichushi; conference abstract | 26 / F | cardiopulmonary arrest | Yes | NR | ABPC/SBT, PIPC/TAZ | Survived |
| 25 | Amatatsu 2023 | Ichushi; Japanese case report | 90 / F | HF, DM, COVID-19 | Yes | NR | PIPC/TAZ, LVFX | Died |
| 26 | Sada 2024 | PubMed/WoS; full article | 68 / F | esophageal cancer; gastric cancer, CVC | Yes | WGS | CMZ | Survived |
| 27 | Sada 2024 | PubMed/WoS; full article | 81 / F | dermatomyositis | Yes | WGS | CTRX | Survived |
| 28 | Sada 2024 | PubMed/WoS; full article | 77 / M | aortic valve regurgitation (aortic valve replacement therapy), end-stage kidney disease, CVC | Yes | WGS | MEPM | Died |
| 29 | Sada 2024 | PubMed/WoS; full article | 53 / M | end-stage kidney disease (kidney transplantation), DM | Yes | WGS | MEPM, MNZ | Survived |
| 30 | Sada 2024 | PubMed/WoS; full article | 19 / F | pulmonary hypertension, CVC | Yes | WGS | VCM | Survived |
| 31 | Kawamoto 2024 | PubMed/WoS; full article | 70s / M | colon cancer, angina pectoris, pancreatic cysts, alcohol use disorder, hypertension, CVC | Yes | WGS | CEZ | Died |

Data were extracted from published reports and conference abstracts when individual case-level information was available. Probiotic exposure was classified as “Yes” when administration of *C. butyricum*, Miya-BM, Miyairi, or a butyric acid bacteria preparation was explicitly described, and as “No” when the report explicitly stated no probiotic exposure. Because microbiological methods and reporting completeness varied across reports, diagnostic certainty should be interpreted cautiously.

* In case 12, *Klebsiella aerogenes* and *Streptococcus anginosus* were co-isolated from blood cultures.

ABPC/SBT, ampicillin/sulbactam; CFPM, cefepime; CMZ, cefmetazole; COPD, chronic obstructive pulmonary disease; CTRX, ceftriaxone; CVC, central venous catheter; DAP, daptomycin; DM, diabetes mellitus; HF, heart failure; LVFX, levofloxacin; MALDI-TOF MS, matrix-assisted laser desorption/ionization time-of-flight mass spectrometry; MCFG, micafungin; MEPM, meropenem; MNZ, metronidazole; NOMI, non-occlusive mesenteric ischemia; NR, not reported; PIPC/TAZ, piperacillin/tazobactam; RAPD, random amplified polymorphic DNA; TEIC, teicoplanin; Tm mapping, melting temperature mapping; VCM, vancomycin; WGS, whole-genome sequencing; WoS, Web of Science

**References**

1. Gardner EM, Kestler M, Beieler A, Belknap RW. Clostridium butyricum sepsis in an injection drug user with an indwelling central venous catheter. J Med Microbiol. 2008;57(Pt 2):236-239. doi:10.1099/jmm.0.47578-0
2. Komori S, Sato A, Otsuka Y, Hosokawa N, Yaegashi M. A case of suspected Clostridium butyricum bacteremia in a patient on probiotics associated with liver cirrhosis and gastric ulcer. Jpn J Chemother [Nihon Kagakuryōhō Gakkai Zasshi]. 2014;62(Suppl A):291. [in Japanese]
3. Kondo S, Nakamura A, Asaoka M, Tagami Y, Ogawa A, Tanaka K. A case of bacteremia caused by Clostridium butyricum during treatment with Miya-BM fine granules. Jpn J Anaerobe Infect Res [Nihon Ken’kiseikin Kansen Shogakkai Zasshi]. 2017;47:34. [in Japanese]
4. Sato Y, Kujirai D, Emoto K, et al. Necrotizing enterocolitis associated with Clostridium butyricum in a Japanese man. Acute Med Surg. 2018;5(2):194-198. Published 2018 Jan 23. doi:10.1002/ams2.329
5. Hiraiwa K, Toyoshima H, Yasuda T, et al. Clostridium butyricum bacteremia developed while taking a butyric acid (Miya-BM tablet). J Gastroenterol Surg [Nihon Shokaki Geka Gakkai Zasshi]. 2020;63:79. [in Japanese]
6. Miyake H, Nakajima T, Miyamoto K, Kato M. A case of Clostridium butyricum bacteremia during probiotic administration. J Jpn Assoc Acute Med [Nihon Kyūkyū Igakkai Zasshi]. 2020;31(11):2127. [in Japanese]
7. Hori K, Nakamura A, Kondo S, et al. A case of Clostridium butyricum bacteremia in a patient receiving Miya-BM granules. Jpn J Anaerobe Infect Res [Nihon Ken’kiseikin Kansen Shogakkai Zasshi]. 2020;50(1):34. [in Japanese]
8. Shimura M, Mizuma M, Nakagawa K, et al. Probiotic-related bacteremia after major hepatectomy for biliary cancer: a report of two cases. Surg Case Rep. 2021;7(1):133. doi:10.1186/s40792-021-01216-5
9. Ogawa J, Asaga T, Saida M, et al. A case of Clostridium butyricum bacteremia during administration of Clostridium butyricum MIYAIRI 588. ICU & CCU [ICU & CCU]. 2021;45:671-674. [in Japanese]
10. Oyabu R, Okamoto K, Kanematsu A, et al. A case of polymicrobial bacteremia including Clostridium butyricum during oral administration of a butyric acid bacteria preparation after living donor liver transplantation. Program/Abstracts Jpn Soc Infect Dis Eastern Branch & Jpn Soc Chemother Eastern Branch Joint Meeting [Nihon Kansensho Gakkai Higashi Nihon Chihoukai Gakujutsu Shūkai / Nihon Kagakuryōhō Gakkai Higashi Nihon Shibu Sōkai]. 2021:169. [in Japanese]
11. Ito R, Kawamura M, Takahashi K, et al. A case of Clostridium butyricum bacteremia associated with probiotic preparation. Jpn J Chemother [Nihon Kagakuryōhō Gakkai Zasshi]. 2022;70(1):161-162. [in Japanese]
12. Watanabe T, Haraki Y, Kusano T, et al. A case of Clostridium butyricum bacteremia in a 2-year-old boy. Jpn J Pediatr Soc [Nihon Shōnika Gakkai Zasshi]. 2022;126(11):1529. [in Japanese]
13. Sanemoto J, Saito H, Nagao E, et al. Two cases of Clostridium bacteremia. J Jpn Soc Perinat Neonat Med [Nihon Shusan Ki Shinseiji Igakkai Zasshi]. 2022;58(Suppl 1):398. [in Japanese]
14. Ishikawa K, Hasegawa R, Shibutani K, et al. Probiotic-related Clostridium butyricum bacteremia: a case report and literature review. Anaerobe. 2023;83:102770. doi:10.1016/j.anaerobe.2023.102770
15. Hayakawa S, Uchiumi T, Ashihara K. A case of acute peritonitis due to Clostridium butyricum in trisomy 21. J Jpn Soc Perinat Neonat Med [Nihon Shusan Ki Shinseiji Igakkai Zasshi]. 2023;59(Suppl 1):P399. [in Japanese]
16. Kusudo N, Yanagita T, Watanabe K, et al. A case of Clostridium butyricum bacteremia caused by administration of Miya-BM during treatment of upper gastrointestinal perforation. J Jpn Surg Assoc [Nihon Rinshō Geka Gakkai Zasshi]. 2023;84(7):1130. [in Japanese]
17. Imado R, Tani J, Takuma K, et al. Cyst infection with Clostridium butyricum following RFA: a case report. Liver [Kanzo]. 2023;64(Suppl 3):A951. [in Japanese]
18. Kojima M, Shimono K, Onishi M. Fatal case of intestinal gas gangrene and Clostridium butyricum bacteremia during probiotic therapy. J Jpn Assoc Acute Med [Nihon Kyūkyū Igakkai Zasshi]. 2023;34(12):821. [in Japanese]
19. Sanada A, Shinjyo K, Uchida Y, Kida K. A case of non-toxin-producing Clostridium butyricum bacteremia. J Jpn Soc Intensive Care Med [Nihon Shūchū Chiryo Igakkai Zasshi]. 2023;30(Suppl 1):S572. [in Japanese]
20. Toyama K, Naganuma Y, Kawamura N, et al. A case in which Clostridium butyricum was detected in blood culture during oral administration of Miya-BM. J Jpn Soc Clin Microbiol [Nihon Rinshō Saikingaku Zasshi]. 2023;34(Suppl 1):353. [in Japanese]
21. Amatatsu N, Momii A, Kawamura H, et al. A case of severe COVID-19 complicated with Clostridium butyricum bacteremia during treatment with a butyric acid bacteria preparation (Miyairi). Jpn J Chemother [Nihon Kagakuryōhō Gakkai Zasshi]. 2023;71(2):277-278. [in Japanese]
22. Sada RM, Matsuo H, Motooka D, et al. Clostridium butyricum Bacteremia Associated with Probiotic Use, Japan. Emerg Infect Dis. 2024;30(4):665-671. doi:10.3201/eid3004.231633
23. Kawamoto Y, Suzuki M, Iwata M, Uehara Y. Probiotics-related Clostridium butyricum bacteraemia after COVID-19, confirmed by whole-genome sequencing. BMJ Case Rep. 2024;17(8):e261578. doi:10.1136/bcr-2024-261578

**Figure S1. Institutional isolate ascertainment process**

Flow diagram showing the ascertainment of institutional blood culture isolates during the study period. Nine isolates were initially reported as *C. butyricum*. In addition, 21 isolates initially reported as *Clostridium* spp. and 79 isolates initially reported as anaerobic Gram-positive bacillus were available as preserved candidate isolates and underwent species-level reevaluation. Seven of these 100 candidate isolates were finally identified as *C. butyricum*, and 93 were excluded because they were not identified as *C. butyricum*. In total, 16 institutional patients with *C. butyricum* isolated from blood cultures were included in the case series.

**Figure S2. PCR ribotyping patterns of institutional *Clostridium butyricum* isolates**

PCR amplification was performed using primers CBISF-CBISR and B2F-CB23R.

N, negative control; P, positive control using probiotics (Miya-BM granule)

**Figure S3. Flow diagram of the literature search and case selection**
