# Supplementary material for: Clinical analysis of 16 patients with Clostridium butyricum isolated from blood cultures: A 10-year single-center case series and review of published cases
Source: New Microbes New Infect. 2026 Jul 30;73:101821. doi: 10.1016/j.nmni.2026.101821 (PMC13453462; doi:10.1016/j.nmni.2026.101821)
Supplement: Supplementary file 2 [file mmc2.docx]

STROBE Statement—checklist of items that should be included in reports of observational studies

Manuscript: Clinical Characteristics of Clostridium butyricum Bacteremia: A 10-Year Single-Center Case Series and Review of Published Cases

|  | Item No. | Recommendation | Page  No. | Relevant text from manuscript |
| --- | --- | --- | --- | --- |
| **Title and abstract** | 1 | (*a*) Indicate the study’s design with a commonly used term in the title or the abstract | 1-2 | Lines 1-2; 4-27 |
|  |  | (*b*) Provide in the abstract an informative and balanced summary of what was done and what was found | 1-2 | Lines 4-27 |
| Introduction | | | |  |
| Background/rationale | 2 | Explain the scientific background and rationale for the investigation being reported | 3-4 | Lines 31-54 |
| Objectives | 3 | State specific objectives, including any prespecified hypotheses | 4 | Lines 49-54 |
| Methods | | | |  |
| Study design | 4 | Present key elements of study design early in the paper | 4 | Lines 56-63 |
| Setting | 5 | Describe the setting, locations, and relevant dates, including periods of recruitment, exposure, follow-up, and data collection | 4, 7 | Lines 57-63; 111-115 |
| Participants | 6 | (*a*) *Cohort study*—Give the eligibility criteria, and the sources and methods of selection of participants. Describe methods of follow-up  *Case-control study*—Give the eligibility criteria, and the sources and methods of case ascertainment and control selection. Give the rationale for the choice of cases and controls  *Cross-sectional study*—Give the eligibility criteria, and the sources and methods of selection of participants | 5, 8 | Lines 65-72; 119-128 |
|  |  | (*b*) *Cohort study*—For matched studies, give matching criteria and number of exposed and unexposed  *Case-control study*—For matched studies, give matching criteria and the number of controls per case | N/A | Not applicable; no matched design or control group |
| Variables | 7 | Clearly define all outcomes, exposures, predictors, potential confounders, and effect modifiers. Give diagnostic criteria, if applicable | 5, 8-9 | Lines 65-81; 129-142 |
| Data sources/ measurement | 8* | For each variable of interest, give sources of data and details of methods of assessment (measurement). Describe comparability of assessment methods if there is more than one group | 5-8 | Lines 65-81; 83-110; 111-128 |
| Bias | 9 | Describe any efforts to address potential sources of bias | 5, 8, 9, 15-16 | Lines 68-71; 119-128; 137-140; 245-265 |
| Study size | 10 | Explain how the study size was arrived at | 4, 9-11 | Lines 57-60; 145-150; 169-174 |

Continued on next page

| Quantitative variables | 11 | Explain how quantitative variables were handled in the analyses. If applicable, describe which groupings were chosen and why | 8-9 | Lines 130-142 |
| --- | --- | --- | --- | --- |
| Statistical methods | 12 | (*a*) Describe all statistical methods, including those used to control for confounding | 8-9 | Lines 129-142 |
|  |  | (*b*) Describe any methods used to examine subgroups and interactions | N/A | Not applicable; no subgroup or interaction analyses were performed |
|  |  | (*c*) Explain how missing data were addressed | 23-24 | Lines 377-378; 389-391; Tables 2 and 3 |
|  |  | (*d*) *Cohort study*—If applicable, explain how loss to follow-up was addressed  *Case-control study*—If applicable, explain how matching of cases and controls was addressed  *Cross-sectional study*—If applicable, describe analytical methods taking account of sampling strategy | N/A | Not applicable; no matched design or formal loss-to-follow-up analysis |
|  |  | (*e*) Describe any sensitivity analyses | N/A | Not applicable; no sensitivity analyses were performed |
| Results | | | | |
| Participants | 13* | (a) Report numbers of individuals at each stage of study—eg numbers potentially eligible, examined for eligibility, confirmed eligible, included in the study, completing follow-up, and analysed | 9-11 | Lines 145-150; 169-174; Supplementary Figure S2 |
|  |  | (b) Give reasons for non-participation at each stage | 8, Suppl. | Lines 121-128; Supplementary Figure S2 |
|  |  | (c) Consider use of a flow diagram | Suppl. | Supplementary Figure S2 |
| Descriptive data | 14* | (a) Give characteristics of study participants (eg demographic, clinical, social) and information on exposures and potential confounders | 10-11 | Lines 154-159; 176-180; Tables 1 and 3 |
|  |  | (b) Indicate number of participants with missing data for each variable of interest | 23-24 | Lines 377-378; 389-391; Tables 1-3 |
|  |  | (c) *Cohort study*—Summarise follow-up time (eg, average and total amount) | N/A | Not applicable; follow-up time was not summarized for this descriptive case series |
| Outcome data | 15* | *Cohort study*—Report numbers of outcome events or summary measures over time | 10-11 | Lines 160-167; 180-184; Tables 2 and 3 |
|  |  | *Case-control study—*Report numbers in each exposure category, or summary measures of exposure | N/A | Not applicable; no case-control design |
|  |  | *Cross-sectional study—*Report numbers of outcome events or summary measures | N/A | Not applicable; no cross-sectional design |
| Main results | 16 | (*a*) Give unadjusted estimates and, if applicable, confounder-adjusted estimates and their precision (eg, 95% confidence interval). Make clear which confounders were adjusted for and why they were included | 11 | Lines 176-184; Table 3 |
|  |  | (*b*) Report category boundaries when continuous variables were categorized | Table 3 | Table 3 (Charlson comorbidity index categories: 0-1, 2-3, >=4) |
|  |  | (*c*) If relevant, consider translating estimates of relative risk into absolute risk for a meaningful time period | N/A | Not applicable; relative risks were not estimated |

Continued on next page

| Other analyses | 17 | Report other analyses done—eg analyses of subgroups and interactions, and sensitivity analyses | 11 | Lines 169-184 |
| --- | --- | --- | --- | --- |
| Discussion | | | | |
| Key results | 18 | Summarise key results with reference to study objectives | 12 | Lines 187-196 |
| Limitations | 19 | Discuss limitations of the study, taking into account sources of potential bias or imprecision. Discuss both direction and magnitude of any potential bias | 15-16 | Lines 245-265 |
| Interpretation | 20 | Give a cautious overall interpretation of results considering objectives, limitations, multiplicity of analyses, results from similar studies, and other relevant evidence | 12-16 | Lines 197-244; 266-271 |
| Generalisability | 21 | Discuss the generalisability (external validity) of the study results | 15-16 | Lines 258-261; 245-265 |
| Other information | |  | | |
| Funding | 22 | Give the source of funding and the role of the funders for the present study and, if applicable, for the original study on which the present article is based | Title page | Title page: Funding, None to declare |

*Give information separately for cases and controls in case-control studies and, if applicable, for exposed and unexposed groups in cohort and cross-sectional studies.

**Note:** An Explanation and Elaboration article discusses each checklist item and gives methodological background and published examples of transparent reporting. The STROBE checklist is best used in conjunction with this article (freely available on the Web sites of PLoS Medicine at http://www.plosmedicine.org/, Annals of Internal Medicine at http://www.annals.org/, and Epidemiology at http://www.epidem.com/). Information on the STROBE Initiative is available at www.strobe-statement.org.
